# Supplementary material for: A yeast surface display platform for characterizing CAR T cell responses to cancer antigens
Source: Nat Commun. 2025 Nov 21;16:10306. doi: 10.1038/s41467-025-65236-7 (PMC12638309; doi:10.1038/s41467-025-65236-7)
Supplement: Supplementary file 2 — Description of Additional Supplementary Files [file 41467_2025_65236_MOESM2_ESM.pdf]

Description of Additional Supplementary Files

Supplementary Data 1: mRNA levels from transcriptome analysis for promoter selection - transcription levels of selected genes of *S. cerevisiae* transcriptome during pheromone response pathway (PRP) activation as an indicator for determining candidate promoter parts.

Supplementary Data 2: Statistical analyses of candidate promoter parts examined by yEGFP - statistical analyses of flow cytometric measurements and fluorescence data (yEGFP) to characterize the candidate promoter parts for the constructed system.

Supplementary Data 3: Pheromone response pathway (PRP) boost in-depth analysis - statistical tests and regressions employed to characterize the observed PRP boost effect and to understand its underlying mechanisms.

Supplementary Data 4: Dose-response curve regressions for candidate promoters - non-linear curve fits for fluorescence data (yEGFP) of the candidate promoters during pathway activation.

Supplementary Data 5: Statistical analyses of CD19 yeast surface display - analyses and statistics of the presentation of CD19 on the yeast cell surface using different designs and systems, e.g. the promoter library and traditional galactose induction, as described by different measurements.

Supplementary Data 6: Statistical analyses of the G protein-coupled receptor (GPCR) library for CD19 display - analyses employed to characterize the GPCRs employed for cognate ligand-based control of CD19 presentation.

Supplementary Data 7: Statistical analyses of co-cultivation conditions - analyses of co-cultivation parameters for the cultivation of yeast and naïve human T cells.

Supplementary Data 8: Statistical analyses of Jurkat NFAT-Luciferase experiments - all statistical analyses used to characterize the Jurkat NFAT-Luciferase activation experiments with CD19 SCASA yeast and NALM6 cancer cells.

Supplementary Data 9: Size comparison of non-cellular antigen platforms - estimation of the surface areas coated with antigen in the conducted experiments to reflect on the difference in the activation levels between antigen-coated microbeads and microtiter plates.

Supplementary Data 10: Statistical analyses of triple-parameter-reporter Jurkat T cell experiments (NFAT, AP-1, NF-kB) - all statistical analyses used to characterize the TPR Jurkat activation experiments with NALM6 cancer cells and CD19 SCASA yeast simulating various cancer conditions via antigen density and target-to-effector cell ratios for two different CARs.

Supplementary Data 11: Responsiveness analyses - analyses to characterize the responsiveness of triple-parameter-reporter Jurkat T cell responses (NFAT, AP-1, NF-kB) to various simulated cancer conditions of antigen density and target-to-effector cell ratios, as modelled by yeast cells, for two different CARs.

Supplementary Data 12: Statistical analyses for comparisons between yeast, microbeads, and microtiter plates - analyses to characterize the performance and differences between the different antigen platforms in the characterization of two different CARs.

Supplementary Data 13: Regressions for comparisons between yeast and microbeads - non-linear regressions to derive dose-response relationships for yeast and microbeads as a function of CD19 antigen density in the activation of two different CARs in triple-parameter-reporter Jurkat T cells.

Supplementary Data 14: Statistical analyses for the activation of an alternative donor-derived CAR T cell product using CD19 SCASA yeast and NALM6 - all statistical analyses employed to characterize various parameters of the activation of the donor-derived CAR T cell product using yeast and cancer cells.

Supplementary Data 15: Primers - all primers employed in this study.

Supplementary Data 16: Parts - all parts used in this study to construct various genes and expression cassettes. The acquisition of parts via PCR amplification and their sources are provided.

Supplementary Data 17: Plasmids - all plasmids employed and constructed in this study.

Supplementary Data 18: Yeast strains - all yeast strains employed and constructed in this study.
