# Supplementary material for: A yeast surface display platform for characterizing CAR T cell responses to cancer antigens
Source: Nat Commun. 2025 Nov 21;16:10306. doi: 10.1038/s41467-025-65236-7 (PMC12638309; doi:10.1038/s41467-025-65236-7)
Supplement: Supplementary file 4 — Reporting Summary [file 41467_2025_65236_MOESM4_ESM.pdf]

nature portfolio

Corresponding author(s): Michael Krogh Jensen, Emil Damgaard Jensen

Last updated by author(s): Sep 5, 2025

Reporting Summary

Nature Portfolio wishes to improve the reproducibility of the work that we publish. This form provides structure for consistency and transparency in reporting. For further information on Nature Portfolio policies, see our Editorial Policies and the Editorial Policy Checklist.

Statistics

For all statistical analyses, confirm that the following items are present in the figure legend, table legend, main text, or Methods section.

n/a	Confirmed	
☐	☒	The exact sample size (n) for each experimental group/condition, given as a discrete number and unit of measurement
☐	☒	A statement on whether measurements were taken from distinct samples or whether the same sample was measured repeatedly
☐	☒	The statistical test(s) used AND whether they are one- or two-sided<br>*Only common tests should be described solely by name; describe more complex techniques in the Methods section.*
☐	☒	A description of all covariates tested
☐	☒	A description of any assumptions or corrections, such as tests of normality and adjustment for multiple comparisons
☐	☒	A full description of the statistical parameters including central tendency (e.g. means) or other basic estimates (e.g. regression coefficient) AND variation (e.g. standard deviation) or associated estimates of uncertainty (e.g. confidence intervals)
☐	☒	For null hypothesis testing, the test statistic (e.g. F, t, r) with confidence intervals, effect sizes, degrees of freedom and P value noted<br>*Give P values as exact values whenever suitable.*
☒	☐	For Bayesian analysis, information on the choice of priors and Markov chain Monte Carlo settings
☒	☐	For hierarchical and complex designs, identification of the appropriate level for tests and full reporting of outcomes
☐	☒	Estimates of effect sizes (e.g. Cohen's d, Pearson's r), indicating how they were calculated

Our web collection on statistics for biologists contains articles on many of the points above.

Software and code

Policy information about availability of computer code

Data collection	Flow cytometer control software:<br>- NovoExpress Software v1.6.2 (Agilent)<br>- CytExpert Acquisition and Analysis Software v2.5 (Beckman Coulter)
Data analysis	FlowJo™ v10.8 Software (BD Life Sciences)<br>GraphPad Prism v9.4.1 (GraphPad Software)<br>Microsoft Excel for Microsoft 365 MSO (Version 2508)

For manuscripts utilizing custom algorithms or software that are central to the research but not yet described in published literature, software must be made available to editors and reviewers. We strongly encourage code deposition in a community repository (e.g. GitHub). See the Nature Portfolio guidelines for submitting code & software for further information.

Data

Policy information about availability of data

All manuscripts must include a data availability statement. This statement should provide the following information, where applicable:

- Accession codes, unique identifiers, or web links for publicly available datasets
- A description of any restrictions on data availability
- For clinical datasets or third party data, please ensure that the statement adheres to our policy

Source data and analyses generated for this study are provided with this paper as a Source Data file and as a Supplementary Tables file, respectively. No datasets deposited in databanks. There are no restrictions on data availability when the study is published.

Research involving human participants, their data, or biological material

Policy information about studies with human participants or human data. See also policy information about sex, gender (identity/presentation), and sexual orientation and race, ethnicity and racism.

Reporting on sex and gender	No such information has been gathered.
Reporting on race, ethnicity, or other socially relevant groupings	No such information has been gathered.
Population characteristics	No such information has been gathered.
Recruitment	Human peripheral blood was obtained from healthy adults after obtaining informed consent (Technical University of Denmark - Rigshospitalet National Hospital approval BC-40). No personal information was gathered, and donors were anonymized for this study. All T cells were derived from the blood of healthy donors collected at the central blood bank at Rigshospitalet (Copenhagen, Denmark).
Ethics oversight	This study was carried out in accordance with the Declaration of Helsinki, and under the agreement: Technical University of Denmark - Rigshospitalet National Hospital approval BC-40.

Note that full information on the approval of the study protocol must also be provided in the manuscript.

Field-specific reporting

Please select the one below that is the best fit for your research. If you are not sure, read the appropriate sections before making your selection.

☒ Life sciences ☐ Behavioural & social sciences ☐ Ecological, evolutionary & environmental sciences

For a reference copy of the document with all sections, see nature.com/documents/nr-reporting-summary-flat.pdf

Life sciences study design

All studies must disclose on these points even when the disclosure is negative.

Sample size	To ensure stable sample populations and capture of potential smaller outlier populations or rare events, the investigated cell populations had a sample size within the range of 13.000 - 1.200.000 cells per biological replicate. A standard of 3 or more biological replicates per tested condition was used, to satisfy the minimal amount of replicates for statistics, while for analyses across conditions employed pooling of such replicates to determine overall behavior. Only one supplementary analysis employed 2 biological replicates (Suppl. Fig. 25). Statistics were calculated on these sample sizes, always testing distributions, normality, and variance. All statistical tests for all results are specified in the Supplementary Tables file.
Data exclusions	No replicates or conditions were excluded from analyses. For flow cytometry data analysis, gating was employed to clean raw data as described and illustrated in Suppl. Fig. 32 and Suppl. Fig. 33.
Replication	There were no issues in replicating the effects seen in the individual experiments. Trial runs were done for experiments to test responses and to get an initial idea of the resolution and range of the behavior to be found in large-scale experiments. Experimental procedures were tested in small-scale before proceeding to large-scale experiments. The reproducibility of the findings enabled such informative trial runs prior to the expensive large-scale experiments.
Randomization	No allocation of samples was done: cells examined or employed originated from a collective source and all conditions were applied to all cell types (e.g. the same isolate of donor-derived CAR T cells was exposed to all conditions).
Blinding	No blinding was needed in this study, because the work involves determining the effects of rationally engineered strains on the same population of immune cells.

Reporting for specific materials, systems and methods

We require information from authors about some types of materials, experimental systems and methods used in many studies. Here, indicate whether each material, system or method listed is relevant to your study. If you are not sure if a list item applies to your research, read the appropriate section before selecting a response.

Materials & experimental systems

n/a	Involved in the study
☐	☒ Antibodies
☐	☒ Eukaryotic cell lines
☒	☐ Palaeontology and archaeology
☒	☐ Animals and other organisms
☒	☐ Clinical data
☒	☐ Dual use research of concern
☒	☐ Plants

Methods

n/a	Involved in the study
☒	☐ ChIP-seq
☐	☒ Flow cytometry
☒	☐ MRI-based neuroimaging

Antibodies

Antibodies used

mouse anti-CD19 (clone: FMC63) (Absolute Antibody, Cat.#Ab00613-2.0), 2.5-5 µg/mL (1:200-1:100)
goat anti-mouse-AF647 (clone: polyclonal) (Thermo Fisher, Cat.#A-21236), 40 µg/mL (1:50)
rabbit anti-HA (clone: RM305) (Thermo Fisher, Cat.#MA5-27915), 2.5 µg/mL (1:400)
goat anti-rabbit-AF488 (clone: polyclonal) (Thermo Fisher, Cat.#A-11008), 10 µg/mL (1:200)
anti-CD3-BV421 (clone: UCHT1) (BD Biosciences, Cat.#562426), dilution1:50
anti-CD69-PE/Cy7 (clone: FN50) (BioLegend, Cat.#310912), dilution 1:150
anti-CD3-perCP (clone: HIT3a) (BioLegend, Cat.#300326), dilution 1:30
anti-c-myc-Dylight488 (clone: 9E10) (Abcam, Cat.#ab117499), dilution 1:40
anti-CD19-BV785 (clone: HIB19) (BioLegend, Cat.#302240), dilution 1:50
anti-CD25-BB700 (clone: M-A251) (BD Biosciences, Cat.#566448), dilution 1:50
anti-CD3-FITC (clone: SK7) (BD Biosciences, Cat.#349201), dilution 1:50

Validation

Validation and citations for the common usage can be found for the individual antibodies on the vendors website. Antibodies were handled and used with regards to the specified recommendations by the suppliers. Antibodies were titrated to find the optimal staining concentration. Specification sources:

Mouse anti-CD19 (clone: FMC63) (Absolute Antibody, Cat.#Ab00613-2.0)
https://absoluteantibody.com/product/anti-cd19-fmc63/Ab00613-2.0_mouse_igg2a/

goat anti-mouse-AF647 (clone: polyclonal) (Thermo Fisher, Cat.#A-21236)
https://www.thermofisher.com/antibody/product/Goat-anti-Mouse-IgG-H-L-Highly-Cross-Adsorbed-Secondary-Antibody-Polyclonal/A-21236

rabbit anti-HA (clone: RM305) (Thermo Fisher, Cat.#MA5-27915)
https://www.thermofisher.com/antibody/product/HA-Tag-Antibody-clone-RM305-Recombinant-Monoclonal/MA5-27915

goat anti-rabbit-AF488 (clone: polyclonal) (Thermo Fisher, Cat.#A-11008)
https://www.thermofisher.com/antibody/product/Goat-anti-Rabbit-IgG-H-L-Cross-Adsorbed-Secondary-Antibody-Polyclonal/A-11008

anti-CD3-BV421 (clone: UCHT1) (BD Biosciences, Cat.#562426)
https://www.bdbiosciences.com/en-eu/products/reagents/flow-cytometry-reagents/research-reagents/single-color-antibodies-ruo/bv421-mouse-anti-human-cd3.562426

anti-CD69-PE/Cy7 (clone: FN50) (BioLegend, Cat.#310912)
https://www.biolegend.com/en-us/soluble-mhc/pe-cyanine7-anti-human-cd69-antibody-1918

anti-CD3-perCP (clone: HIT3a) (BioLegend, Cat.#300326)
https://www.biolegend.com/nl-be/cell-health/percp-anti-human-cd3-antibody-5612

anti-c-myc-Dylight488 (clone: 9E10) (Abcam, Cat.#ab117499)
https://www.abcam.com/en-dk/products/primary-antibodies/fitc-myc-tag-antibody-9e10-ab117599

anti-CD19-BV785 (clone: HIB19) (BioLegend, Cat.#302240)
https://www.biolegend.com/en-us/products/brilliant-violet-785-anti-human-cd19-antibody-7967

anti-CD25-BB700 (clone: M-A251) (BD Biosciences, Cat.#566448)
https://www.bdbiosciences.com/en-dk/products/reagents/flow-cytometry-reagents/research-reagents/single-color-antibodies-ruo/bb700-mouse-anti-human-cd25.566448

anti-CD3-FITC (clone: SK7) (BD Biosciences, Cat.#349201)
https://www.bdbiosciences.com/en-us/products/reagents/flow-cytometry-reagents/clinical-diagnostics/single-color-antibodies-asr-ivd-ce-ivd/cd3-fitc.349201

Eukaryotic cell lines

Policy information about cell lines and Sex and Gender in Research

Cell line source(s)	NALM6 B cell precursor leukemia cell line: DSMZ, no.: ACC 128.<br>HEK293 cells: ATCC, 293T Cat.#CRL-3216.<br>Jurkat NFAT-Luc: Nordic BioSite, Cat.#BPS-60621.<br>CAR Jurkat NFAT-Luc: Nordic BioSite, Cat.#BPS-60621.<br>Triple parameter T cell reporter cell line (Jutz et al. 2016)<br>Primary T cells - from peripheral blood (See: "Research involving human participants, their data, or biological material").<br>CAR T cells generated from primary T cells (See: "Research involving human participants, their data, or biological material").
Authentication	Cell lines has not been further authenticated than performed by the supplier.
Mycoplasma contamination	All our cell lines are routinely tested and have tested negative for Mycoplasma before use.
Commonly misidentified lines (See ICLAC register)	No commonly misidentified cell lines were used.

Plants

Seed stocks	N/A
Novel plant genotypes	N/A
Authentication	N/A

Flow Cytometry

Plots

Confirm that:

☒ The axis labels state the marker and fluorochrome used (e.g. CD4-FITC).

☒ The axis scales are clearly visible. Include numbers along axes only for bottom left plot of group (a 'group' is an analysis of identical markers).

☒ All plots are contour plots with outliers or pseudocolor plots.

☒ A numerical value for number of cells or percentage (with statistics) is provided.

Methodology

Sample preparation	For yeast cell staining, cells from a culture were pelleted (800g, 3 min., 5°C), washed twice in 150 µL ice-cold PBSA (1X PBS with 1 g/L bovine serum albumin (Sigma-Aldrich, Cat.#A4503)), then resuspended and incubated in 50 µL primary antibody mix (darkness, 30 min., on ice, 200 r.p.m.). Hereafter, the procedure was repeated for the secondary antibody mix. Finally, cells were pelleted and washed twice in 150 µL ice-cold PBSA, whereafter they were resuspended in 150 µL ice-cold PBSA for flow cytometry.<br>For staining of co-cultures of T cells and yeast, co-cultures were first pelleted (300g, 5 min., 5°C) to then carefully aspirate the media, whereafter each replicate was resuspended in 50 µL staining master mix and incubated (darkness, 30 min., on ice). Hereafter, the cells were pelleted (300g, 5 min., 5°C), washed twice with 150 µL ice-cold Cell Staining Buffer, and then resuspended in 100 µL ice-cold Cell Staining Buffer for flow cytometry.<br><br>The Methods section specifies this process for each experiment.
Instrument	- NovoCyte Quanteon 4025 Flow Cytometer System with a NovoSampler Q System (Agilent)<br>- CytoFLEX-S (V-B-Y-R) instrument (Beckman Coulter)
Software	Flow cytometer control software:<br>- NovoExpress Software v1.6.2 (Agilent)<br>- CytExpert Acquisition and Analysis Software v2.5 (Beckman Coulter)

Gating and compensation:
- FlowJo™ v10.8 Software (BD Life Sciences)

Cell population abundance

Yeast yEGFP experiments:
20.000 events collected per replicate. Post-gating: >18.000 events per replicate.

Yeast CD19 YSD experiments:
50.000 events collected per replicate. Post-gating: >44.000 events per replicate.

Examination of viability and proliferation of T cells in yeast co-cultures:
Volume-based: 13.000-100.000 events per replicate. Post-gating: 3.000-7.000 T cells (CD3+) per replicate.

Activation of donor-derived CAR T cells using NALM6 and SCASA yeast:
Yeast co-cultivations (volume-based):
110.000-1.200.000 events per replicate, Post-gating: 25.000-70.000 T cells (CD3+) per replicate.
NALM6 co-cultivations (volume-based):
60.000-500.000 events per replicate, Post-gating: 30.000-80.000 T cells (CD3+) per replicate.

Post-cryopreservation activation of donor-derived CAR T cells using SCASA yeast:
120.000 events collected per replicate. Post-gating: 10.000-30.000 T cells (CD3+) per replicate.

These numbers for each gate are exemplified in the gating strategies shown in Suppl. Fig. 32 and Suppl. Fig. 33.

Gating strategy

Gating and compensation were done using FlowJo™ v10.8.1 Software (BD Life Sciences). Gating strategies employed fluorescence minus one (FMO) controls, as well as the employment of negative controls where possible for determining true positive signals (e.g. yEGFP+, CFSE+).

Gating strategy for yEGFP experiments: First singlets were gated ('Singlets': SSC-A vs. SSC-H), then yeast cells were gated for removal of cellular debris and non-cellular events ('Yeast cells': SSC-A vs. FSC-A), then dying or non-responding yeast cells were removed through a NOT-gate, via their distinct morphology and increased red autofluorescence ('Non-responders': Y615-H vs. FSC-A), leaving responsive yeast cells for detection of yEGFP and morphological changes ('Responders'). The example is PMFA1-yEGFP stimulated with 0.1 µM α-factor.

Gating strategy for CD19 YSD: First yeast cells were gated by a NOT-gate removing cellular debris and non-cellular events ('Yeast': SSC-H vs. FSC-H), then singlets were gated by a NOT-gate ('Singlets': SSC-A vs. SSC-H). The example is PFUS1-CD19 stimulated with 0.1 µM α-factor.

Gating for examination of viability and proliferation of T cells in yeast co-cultures: First singlets were gated ('Singlets': SSC-A vs. SSC-H), then for the gating of T cells, intact cells were gated for removal of cellular debris and non-cellular events ('Intact cells': FSC-H vs. SSC-H), which were then gated for their expression of CD3 ('T cells (CD3+)': Comp-V445-H vs. SSC-H). T cells were then gated for CFSE proliferation dye staining ('T cells (CFSE+)': Comp-B525-H vs. FSC-H), and dead and alive cells were identified based on Near-IR (NIR) viability dye ('Dead (NIR+)' / 'Alive (NIR-)': Comp-R780-H vs. FSC-H). For yeast cells, yeast was first identified based on morphological traits ('Yeast': FSC-H vs. SSC-H), then on their lack of CD3 expression ('Yeast (CD3-)': Comp-V445-H vs. FSC-H), and finally their lack of CFSE staining ('Yeast (-CFSE)': Comp-B525-H vs. FSC-H). The example is co-cultivation of T cells with 1.0x yeast, at 0 µM alpha-factor, on Day 5 (96 hrs).

Gating strategy for activation of donor-derived CAR T cells using NALM6: First singlets were gated ('Singlets': SSC-A vs. SSC-H), then for separation of NALM6, the GFP expressing population was gated ('NALM6 (GFPHi+)': FL1-A-B525/40 vs FL2-A-B690/50) and further gated by size ('Normal size NALM6': FSC-H vs. FL9-A-V780/60). For the separation of T cells, a NOT-gate was employed for the NALM6 population ('not NALM6 (GFPHi+)': FL1-A-B525/40 vs FL2-A-B690/50). Then the alive population was gated by a Zombie Violet (ZV) stain ('Alive (ZV-)': FL6-A_V450/45 vs FSC-H). T cells were then identified by CD3 expression ('T cells (CD3+)': FSC-H vs. FL2-A-B690/50), and from here CAR-expressing cells were identified through myc-tag staining ('CAR+ T cells (myc+)': FSC-H vs. FL1-A-B525/40), and non-expressing T cells through a NOT-gate ('CAR- T cells (myc-)': FSC-H vs. FL1-A-B525/40). Activated cells were identified by a CD69 stain ('Activated (CD69+)': FL13-A-Y780/60 vs. FL2-H-B690/50). The same strategy was employed for the CTRL T cell culture. The example is a co-cultivation of CAR T cells with 5.0x NALM6.

Gating strategy for activation of donor-derived CAR T cells using SCASA yeast: First singlets were gated ('Singlets': SSC-A vs. SSC-H), then the alive population was gated by a Zombie Violet (ZV) stain ('Alive (ZV-)': FSC-H vs. FL6-A-V450/45). Hereafter, yeast cells ('Yeast': FSC-H vs. SSC-H) could be separated from human cells ('Not yeast cells': FSC-H vs. SSC-H) through morphological traits. T cells were then identified via CD3 expression ('T cells (CD3+)': FSC-H vs. FL2-A-B690/50), and from here CAR-expressing cells were identified through myc-tag staining ('CAR+ T cells (myc+)': FSC-H vs. FL1-A-B525/40), and non-expressing T cells through a NOT-gate ('CAR- T cells (myc-)'). Activated cells were identified by a CD69 stain ('Activated (CD69 +)': FL13-A-Y780/60 vs. FL2-H-B690/50). Yeast cells were further identified from the lack of CD3 expression ('Yeast (CD3-)': FL2-A-B690/50 vs. SSC-H) for the investigation of CD19 levels. The same strategy was employed for the CTRL T cell culture and the negative control yeast (PPGK1-Empty). The example is a co-cultivation of CAR T cells with 1.0x PPGK1-CD19 SCASA yeast.

Gating strategy for post-cryopreservation activation of donor-derived CAR T cells using SCASA yeast: First the alive population was gated by a Zombie Violet (ZV) stain ('Alive (ZV-)': FSC-H vs. V445-H), then T cells were identified by CD3 expression ('T cells (CD3+)': B525-H vs. FSC-H), and lastly singlets were gated ('Single cells': SSC-H vs SSC-A) for the examination of CD69 and CD25 expression. The same strategy was employed for the other SCASA yeast strains and LAC control. The example is a co-cultivation of CAR T cells with PTDH3-CD19 SCASA yeast (0.3x) without α-factor.

☒ Tick this box to confirm that a figure exemplifying the gating strategy is provided in the Supplementary Information.
